# Exploring contemporary public perceptions of historical redlining practices in the United States

**DOI:** 10.1007/s43762-026-00237-w

**Published:** 2026-01-23

**Authors:** Yujian Lu, Xi Gong, Guiming Zhang, Christopher P. Brown, Yolanda C. Lin, Yan Lin

**Affiliations:** 1 Department of Geography & Environmental Studies, University of New Mexico, Albuquerque, NM 87131 USA; 2 UNM Center for the Advancement of Spatial Informatics Research and Education (ASPIRE), University of New Mexico, Albuquerque, NM 87131 USA; 3 Department of Biobehavioral Health, The Pennsylvania State University, University Park, PA 16802 USA; 4 Institute for Computational and Data Sciences (ICDS), The Pennsylvania State University, University Park, PA 16802 USA; 5 Department of Geography & the Environment, University of Denver, Denver, CO 80210 USA; 6 Department of Geography & Environmental Studies, New Mexico State University, Las Cruces, NM 88011 USA; 7 Department of Geography, The Pennsylvania State University, University Park, PA 16802 USA; 8 Social Science Research Institute (SSRI), The Pennsylvania State University, University Park, PA 16802 USA

**Keywords:** Redlining, Social media, GIS, Multiscale geographically weighted regression (MGWR), Public perceptions

## Abstract

Redlining is a discriminatory practice of systematically denying loans or mortgages to residents in specific neighborhoods based on racial or ethnical composition. In current literature research, there is a lack of understanding of the public perceptions of impacts of historical redlining practices at large geographic scales. Although some social groups and organizations conducted surveys or interviews to obtain public perceptions of it on small groups of people in certain areas, our knowledge of the impacts of redlining is limited and may reflect bias. This study used geotagged tweets from 2011 to 2023 to investigate public perceptions of redlining practices in U.S. counties. Multiscale geographically weighted regression (MGWR) was performed to explore both spatial heterogeneity and varying scales of associations between percentage of redlining-related geotagged tweets with negative sentiment and potential explanatory shaping factors in U.S. counties. Counties with a higher average household size, a higher percentage of people aged 45+, a lower homeownership rate, and a higher mobile home percentage have a significant association nationwide with more negative-sentiment expression in redlining-related tweets. However, counties with a lower insurance coverage are less likely to express negative sentiment in redlining-related tweets in some eastern U.S. counties, indicating a local significant association. The findings help people better understand the relationship between public perceptions of redlining practices and potential shaping factors. This study’s methodology can also be applied to investigate public perspectives or perceptions on other controversial social topics.

## Introduction

Due to the Great Depression in the 1930s, the United States experienced high unemployment rates and widespread foreclosures (Hillier, 2003). To provide assistance to homeowners and stimulate the economy, the Home Owners' Loan Corporation (HOLC) was established by the U.S. federal government as a part of the “New Deal” program (Mendez-Carbajo, 2021; United States Congress, 1933). It aimed to help homeowners refinance their mortgages at a lower interest rate with longer repayment periods to prevent foreclosure (Harriss, 1951). To evaluate the post-refinancing investment risk in all big cities across the whole country, residential security maps were created by HOLC to appraise all neighborhoods in more than 200 U.S. cities (Hillier, 2003; Mendez-Carbajo, 2021; Nelson et al., 2020; United States Congress, 1933). The risk associated with mortgage investments was depicted in four hierarchical categories with color coding: green represents the highest grade of “A”, which was considered "best" and indicates the lowest investment risk for all mortgage lenders; blue represents grade of "B", which was considered "still desirable"; yellow represents grade of "C" and "definitely declining"; and red represents the lowest grade of "D", which indicates "hazardous" and the highest investment risk for lenders (Hillier, 2005a; Nelson et al., 2020). The term “redlining” is derived from mapping the neighborhoods with the highest risk with red color-coding on HOLC security maps (Cornell Law School, 2023). The HOLC security maps not only indicate the grade in each neighborhood in more than 200 cities, but also indicate the conditions of housing stocks and composition of race/ethnicity. Neighborhoods with a grade of "D" were characterized by older housing stocks, and predominantly inhabited by African Americans, Jews, and immigrants; while neighborhoods with a grade of "A" were characterized by newer housing stocks and a higher presence of White U.S.-born people (Hillier, 2005a; Locke et al., 2021; Rothstein, 2017). Through consideration of racial composition as an indicator of perceived value in long-term appraisals of those neighborhoods, race has been formally integrated into the appraisal process, resulting in long standing structural racism in residential housing markets in the United States (Hillier, 2005a; Rothstein, 2017).

Historical redlining practices (hereafter referred to as redlining practices) were identified to have contributed to present-day residential segregation in U.S. cities, further limiting access to social resources and impairing social mobility for communities of color. These barriers include restricted access to high-quality education, well-maintained community facilities, higher household median incomes, and better employment opportunities (Collins & Margo, 2000; Korver-Glenn, 2021). Most existing research focuses on the long-term adverse impact of redlining practices on socioeconomic factors, environmental factors, health outcomes, and urban facilities in those formerly HOLC-graded neighborhoods. Examples of this latter impact are formerly redlined neighborhoods suffering from concentration of poverty (Rutan & Glass, 2018), having less tree canopy (Locke et al., 2021), being more likely to be exposed to COVID-19 risk factors and air pollution (Lane et al., 2022; Richardson et al., 2020). These areas also have a significantly higher rate of incomplete or inadequate plumbing in residential structures (Sterling et al., 2023). In addition, redlining practices have been closely associated with reduced homeownership rates and the suppression of intergenerational wealth accumulation, particularly among marginalized communities. Mendez-Carbajo (2021) found that residents of redlined neighborhoods often lacked the financial resources to purchase homes or maintain existing properties, which hindered their ability to attain or sustain homeownership and accumulate wealth at the same rates as those in non-redlined areas. And he also mentioned that homes in redlined neighborhoods typically failed to appreciate in value, limiting residents’ ability to accumulate wealth, invest in education, or pass on assets to future generations. Consequently, redlining practices significantly hinder the social and economic mobility of affected communities.

While previous studies document crucial insights into systematic inequities due to redlining practices, they often overlook the public perceptions of this historical legacy. Understanding how the public perceives redlining practices is essential, because it shapes social awareness, affects societal discourse, and may drive social reform and policy development aiming to address the legacy of redlining practices. Therefore, it is necessary to integrate public perceptions into redlining-related research to foster a more comprehensive understanding of the impact of redlining practices.

Currently, only some social groups and organizations investigated public perceptions of redlining practices using traditional data collection methods including oral history, life stories, interviews, and surveys. For instance, Tongue (2020) recalled that he and his grandfather faced extremely limited lending opportunities when buying a house outside of redlined areas. MacKenzie (2019) interviewed Dr. Brittany Lewis and they explored the history of the redlining practices, racism, and discrimination in housing policy and practice, emphasizing that the decision-makers did not address racial inequity. In 2018, Merlin Rainwater was interviewed by Mahmoud (2018), they found that many residents in Seattle still suffer from the impact of restrictive covenant redlining on the city. On May 10th -12th, 2022, a public survey was conducted by YouGovAmerica to gauge public perceptions of the American housing market and redlining practices (Orth, 2022). More than half of responders felt that redlining practices occur somewhat or very often, and they opposed this historical policy, especially for Black responders with 63% of them sharing the same perspective. Respondents also approved the government’s efforts to increase regulation to prevent redlining practices (Orth, 2022). Lindsey (1998) also found the perceptions of lending determination vary by race based on survey results.

However, it is important to note that oral history, life stories, interviews, and surveys may introduce bias due to the limited sample size ranging from a few to 1000 participants, which only covers a small number of people or small areas at a particular period. In other words, there is a lack of understanding of the public perceptions spanning a large geographical region. This lack of understanding provides limited insight into perspectives of redlining practices from survivors of such practices (Korver-Glenn, 2021; Orth, 2022). Compared with conventional data collection methods such as surveys or interviews, social media data has several advantages: high volume, low cost, large coverage, and real-time (Rizwan et al., 2020). Therefore, exploring social media data can improve on conventional data collection methods and be a valuable subjective data source for understanding public perspectives on many issues. This study would bridge the literature gap to help people and policymakers gain a better understanding of the public perceptions of redlining practices through exploring social media data.

Social media is a group of Internet-based applications, and it constructs a virtual environment for users to generate and share information (Kaplan & Haenlein, 2010; Kietzmann et al., 2011). With the popularity of social media, a large group of users creates digital footprints online continuously. Some social media platforms, such as Twitter (now known as “X”, hereafter referred to as X), Facebook, and Instagram, also allow geo-referenced sharing of information (Gong & Yang, 2020). Therefore, the emerging of social media data provides us with a valuable opportunity to understand the spatial–temporal patterns of public social behavior and perspectives towards certain controversial social issues at a large geographical scale (Gong & Wang, 2021; Liu et al., 2021). Among social media platforms, X is popular due to its short messaging (up to 280 characters), directed following, quoting, and retweeting features; these qualities can facilitate faster dissemination of information and better interaction between users (Gong & Lane, 2020; Gong & Ye, 2021; Huberman et al., 2008; Kwak et al., 2010). Consequently, this study examines X data/tweets (hereafter referred to as tweets) to obtain a large sample of public perceptions of redlining practices in the United States.

Two research questions would be addressed by this study. First, which shaping factors, including sociodemographic, economic, and housing characteristics, significantly influence the public perceptions of redlining practices? Second, what are the geographical scales and spatial variations in the influence of shaping factors on public perceptions? By examining these two questions, we can understand the impacts of factors that have the potential to shape the social dynamics underlying these perceptions from a geographical lens. Answering these questions can offer valuable insights into collective awareness of the long-term impacts of redlining practices. Furthermore, through deepening understanding of these effects, this research can serve as a foundation for fostering public engagement in addressing the historical legacy of redlining practices, ultimately contributing to the development of more inclusive and equitable cities.

## Data

We collected tweets to investigate the public perceptions of redlining practices, alongside census data to identify potential shaping factors influencing these perceptions.

### Data for public perceptions

We collected tweets related to redlining practices and their impacts by using X Application Programming Interfaces for Academic Research (APIs V.2) from Mar 1st, 2011, to Mar 31st, 2023, to examine tweets in the United States (Twitter Inc., 2022). The start date was chosen because the number of tweets related to redlining practices and their impacts prior to March 2011 was extremely limited—fewer than 100 each year—based on platform availability. The keywords and keyword combinations chosen and summarized from current literature in Table 1 were used to filter the related tweets. While direct mentions of redlining practices are relatively limited on social media, the impacts of redlining practices—such as *housing segregation*—were also included to improve the visibility of discussions on redlining practices, recognizing redlining practices are often referred as a reason for current housing segregation (Gerken et al., 2023), and two terms are frequently used interchangeably or in close association in social media discussions. Furthermore, incorporating both terms allows for a more comprehensive exploration of public perceptions of redlining practices and their potential impacts on social media. Every collected tweet record has its username, ID, time created, and full text.

**Table 1:** Keywords and keyword combinations for filtering the redlining-related tweets

Keyword combinations	Keyword combinations	Keyword combinations
redlining	lending discrimination	Jim Crow^c^
redlined	residential segregation	housing crisis
Home Owner’s Loan Corporate	place-based inequality	mortgage, housing
HOLC	urban renewal	exclusive, housing
New Deal, Roosevelt	Fair Housing Act	exclusive, neighborhoods
greenlined	FHA, Underwriting Manual^b^	exclusive, mortgage
white flight^a^	restrictive covenant, house	exclusive, property
lending bias	restrictive covenant, home	exclusive, district
racist rubric	Racial discrimination, housing	housing, justice
housing segregation	housing insecurity	racial segregation

^a^White Flight refers to white Americans' historical migration from urban areas to suburbs, primarily during the mid-twentieth century. Several factors contributed to white flight, including the desire for larger homes, better schools, and improved infrastructure in suburban areas. However, race was often a central factor, with many whites fearing the negative effects of racial integration on property values and social status (Planetizen, 2025)

^b^Underwriting Manual: In 1935, Underwriting Manual was issued by the Federal Housing Administration (FHA), which described procedures and policies for determining mortgage insurance eligibility based on risk rating and valuation considerations, but it stated that “If a neighborhood is to retain stability it is necessary that properties shall continue to be occupied by the same social and racial classes. A change in social or racial occupancy generally leads to instability and a reduction in values” (Federal Housing Administration, 1935)

^c^Jim Crow was a system of laws that kept Black and White Americans separated in many public places, like schools, transportation, and restaurants, mainly in the Southern United States from the late 1800s to the mid-1900s (Pilgrim, 2012)

**Fig. 1:**
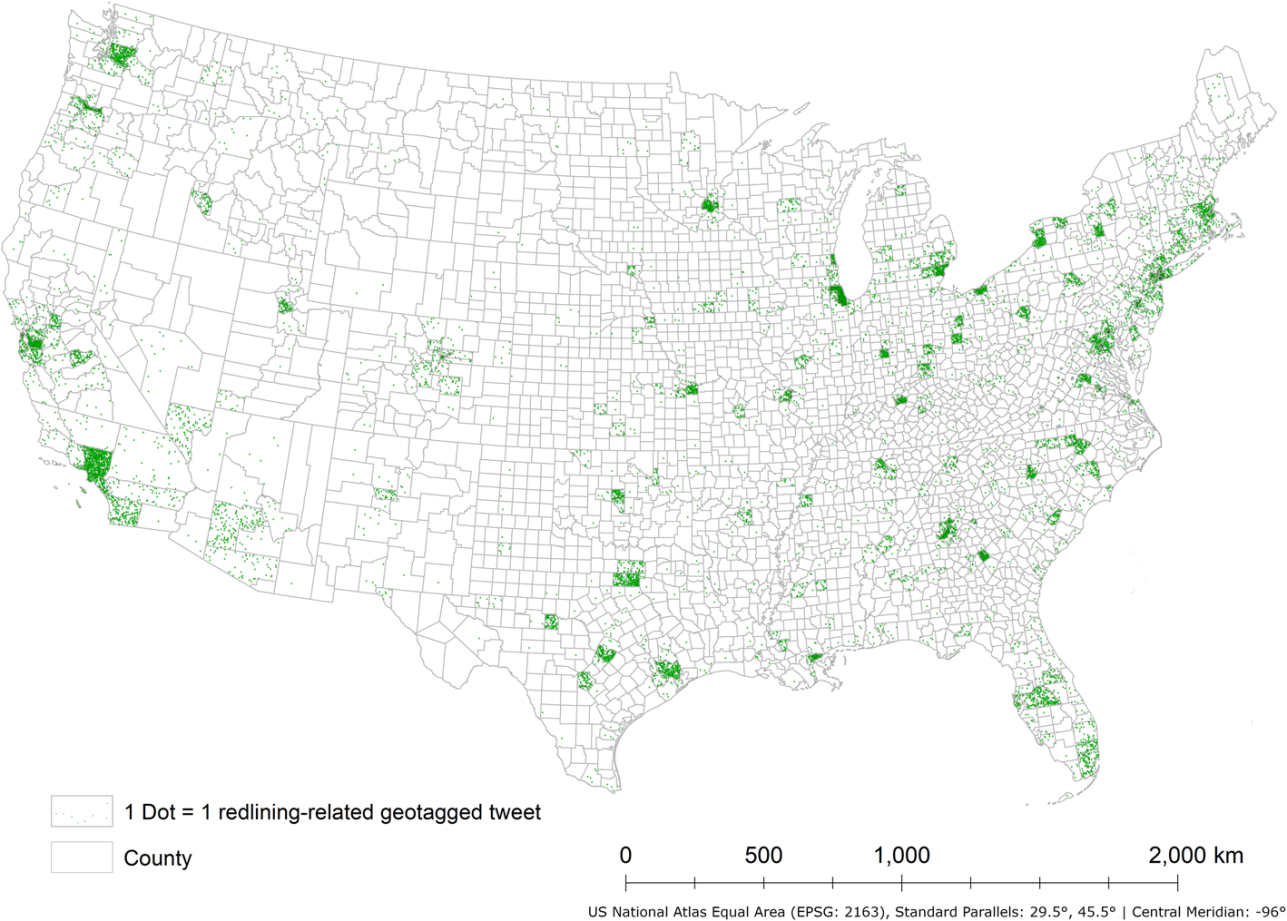
The distribution of redlining-related geotagged tweets in the contiguous U.S. counties from Mar 1st, 2011, to Mar 31.^st^, 2023

To analyze the spatial heterogeneity and spatial variation of those tweets, we examined the tweets with geotags, such as coordinates, street address, city name, etc. (Twitter Developer Platform, 2020). The tweets with street address or city name were geocoded using ArcGIS 10.8.1 (ESRI, 2023). Consequently, 18,938 geotagged tweets were pre-processed for further analysis in the contiguous United States (Fig. 1).

### Data for potential shaping factors

Most previous research focuses on sociodemographic and economic factors as potential shaping factors for redlining practices, but our research also includes housing characteristics to explore the influence of those three categories on public perceptions of redlining practices. Therefore, we collected data based on those three categories for each U.S. county in 2020 from the U.S. Census Bureau (2022). Table 2 shows those three categories and potential shaping factors in each category.

**Table 2:** Potential shaping factors for redlining practices in three categories

Category	Potential shaping factors
Sociodemographic	Age distribution (percentages of population < 18, 18–44, and 45+)
	Sex ratio (male-to-female ratio)
	Percentages of different race/ethnicity groups (non-Hispanic White, Hispanic and Latinos, and African Americans)
Economic	Household median income
	Educational attainment (percentage of people aged 25 or above with a bachelor’s degree)
	Uninsured population percentage
Housing characteristics	Homeownership rate
	Average household size
	Percentage of crowded households^a^
	Mobile home percentage

^a^Crowded households describe living conditions where the number of people in a home exceeds the amount of space available for comfortable living

## Methods

Sentiment analysis was applied to determine the sentiment expressed in each geotagged tweet, while two regression models were utilized to assess the associations between public perceptions of redlining practices and potential shaping factors at both global-scale and local-scale.

### Sentiment analysis

To better understand the sentiment of each geotagged tweet, we first removed the links, special characters such as comma, “@”, and “#”, and unmeaningful words such as “the”, “in”, and “on” in those tweets. Secondly, all words in each geotagged tweet were converted to their root forms. Thirdly, the BERTweet-based sentiment analysis model (Nguyen et al., 2020; Pérez et al., 2021) was applied to each redlining-related geotagged tweet to detect its sentiment. BERTweet is a language model pre-trained based on 850 million English tweets, including 845 million streamed tweets from Jan. 2012 to Aug. 2019 and 5 million tweets related to COVID-19 pandemic (Nguyen et al., 2020). This model can be used to predict the sentiment of input English tweets as either positive, neutral, or negative with a higher accuracy. Sentiment analysis generates 7,697 negative geotagged tweets, 1,582 positive geotagged tweets, and 9,659 neutral geotagged tweets, which account for 40.64%, 8.35%, and 51.00% of all geotagged tweets, respectively. In our analysis, we used the percentage of redlining-related geotagged tweets with negative sentiment to represent the public perceptions of redlining.

### Regression analysis

Percentage of redlining-related geotagged tweets with negative sentiment in each county was used as dependent variable. VIF (variance inflation factor) test was performed on all potential explanatory shaping factors as mentioned in section “Data for potential shaping factors” to detect multicollinearity (Shrestha, 2020). After removing the shaping factors from the same category with higher multicollinearity, the 12 remaining explanatory shaping factors are: percentage of people aged 18–44 and aged 45+, sex ratio (male-to-female ratio), percentage of Hispanic and Latinos, percentage of African Americans, household median income, educational attainment, uninsured population percentage, homeownership rate, average household size, percentage of crowded households, and mobile home percentage.

Two different regression models were applied to detect the associations between the 12 potential explanatory shaping factors and percentage of redlining-related geotagged tweets with negative sentiment across U.S. counties. The ordinary least squares (OLS) was used first to examine the global-scale association between contributing shaping factors and percentage of geotagged tweets with negative sentiment, offering clear insights into global-scale relationships. However, this model fails to examine the spatial heterogeneity and associations at different spatial scales (such as national, state, or local scales). Unlike OLS regression, which assumes parameter estimates remain constant across geographic space, Multiscale Geographically Weighted Regression (MGWR) allows different regions to have unique parameter values, capturing spatial heterogeneity (how relationships change across locations) and spatial variation at different scales (local, regional, and global differences) (Fotheringham et al., 2017). Therefore, the MGWR model was used to analyze those spatial patterns. The formulation of the MGWR model is:

1 $${y}_{i}= {\sum }_{j=0}^{m}{\beta }_{bwj}\left({u}_{i},{v}_{i}\right){x}_{ij}+{\varepsilon }_{i}$$

Where ${y}_{i}$ represents percentage of redlining-related geotagged tweets with negative sentiment for county *i*, $bwj$ in ${\beta }_{bwj}$ stands for the bandwidth of $j$ th potential shaping factor, $\left({u}_{i}, {v}_{i}\right)$ denotes the centroid coordinate of county *i*, ${x}_{ij}$ is the $j$ th potential shaping factor for county *i*, ${\beta }_{bwj}\left({u}_{i},{ v}_{i}\right)$ is the coefficient of $j$ th potential shaping factor, and ${\varepsilon }_{i}$ is the error term (Fotheringham et al., 2017).

The parameter of bandwidth (*bwj*) represents the number of nearest counties, from which the data was weighted for calculation of each local regression (Li & Fotheringham, 2020; Oshan et al., 2019). And its coefficients can be computed to illustrate how the relationship between public perceptions and each explanatory shaping factor varies across different spatial scales (Li & Fotheringham, 2020; Oshan et al., 2019). A shaping factor with a small bandwidth is considered to have a more localized influence on public perceptions, while one with a large bandwidth reflects regional-scale or global-scale impacts (Li & Fotheringham, 2020; Oshan et al., 2019). For each explanatory shaping factor, the optimal bandwidth and its corresponding association coefficients in different areas can be obtained by performing MGWR to reflect spatial scale and spatial variation. The model was calibrated using a bi-square spatial kernel weighting scheme, and its performance in optimization was evaluated using a corrected Akaike Information Criterion (AICc), in which a lower AIC value indicates a better model fit while a higher AIC value indicates a less favorable model, potentially overfitting or failing to explain the data well (Li & Fotheringham, 2020; Oshan et al., 2019).

## Results and discussion

**Fig. 2:**
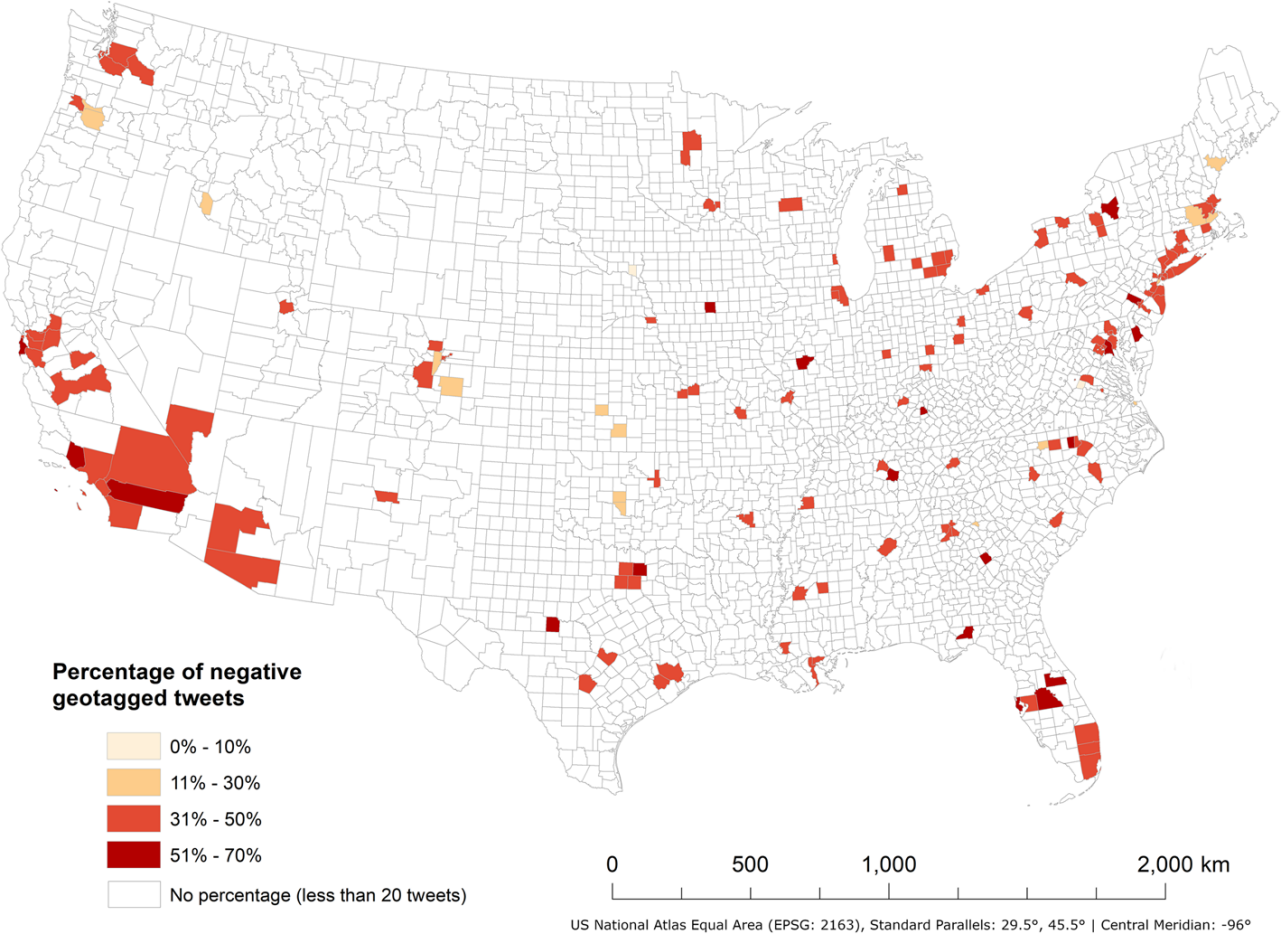
Percentage of redlining-related geotagged tweets with negative sentiment in contiguous U.S. counties with 20 or more redlining-related geotagged tweets from Mar 1st, 2011, to Mar 31st, 2023

To ensure the statistical robustness of this research, only 157 counties in the contiguous United States with 20 or more redlining-related geotagged tweets were selected for further analysis. This threshold reflects a tradeoff between maximizing geographic coverage and maintaining sufficient tweet volume per county to support reliable geospatial analysis. There are 15,945 geotagged tweets in total in those 157 counties (18,938 geotagged tweets in original pool). Three counties have more than 700 geotagged tweets, which are Cook County, Illinois (925), Los Angeles County, California (913), and New York County, New York (739). After sentiment analysis for all geotagged tweets using BERTweet-based sentiment analysis model was conducted, 40.7% of geotagged tweets are identified with negative sentiment (Fig. 2). The percentage of geotagged tweets with negative sentiment on average is 40.4% in those counties. 24 counties have over 50% of redlining-related geotagged tweets expressing negative sentiment, while five counties exceed 60%, including Montgomery County, Pennsylvania (70.0%), Collin County, Texas (67.5%), Kent County, Delaware (63.0%), Ventura County, California (62.1%), and Mercer County, Kentucky (61.3%).

Table 3 lists the parameter coefficients and *p*-values for all potential explanatory shaping factors associated with public perceptions of redlining practices based on OLS regression analysis. Average household size indicates a statistically significant positive association with public perceptions of redlining practices. With the increase in average household size in a specific county, percentage of redlining-related geotagged tweets with negative sentiment also increases. For all other 11 shaping factors, no significant associations are observed. But those explanatory shaping factors may show significant associations at regional or local geographical scales, the MGWR model was used to explore the spatial variations of all 12 shaping factors across different geographical locations and scales.

**Table 3:** The results of OLS regression analysis for associations between percentage of redlining-related geotagged tweets with negative sentiment and potential explanatory shaping factors in contiguous U.S. counties from Mar 1st, 2011, to Mar 31st, 2023

Explanatory shaping factors	Coefficient	*p*-value
Percentage of people aged 18 to 44	-0.235	0.301
Percentage of people aged 45 and above	0.224	0.229
Sex ratio (male-to-female ratio)	0.087	0.501
Percentage of Hispanics and Latinos	-0.084	0.611
Percentage of African Americans	0.036	0.766
Household median income	-0.219	0.377
Percentage of people aged 25 or above with a bachelor’s degree	0.283	0.234
Percentage of people without health insurance	-0.037	0.729
Homeownership rate	-0.412	0.126
Average household size	0.475*	0.026
Percentage of crowded households	-0.186	0.347
Percentage of mobile home	0.200	0.068

^*^p < *0.05*

**Fig. 3:**
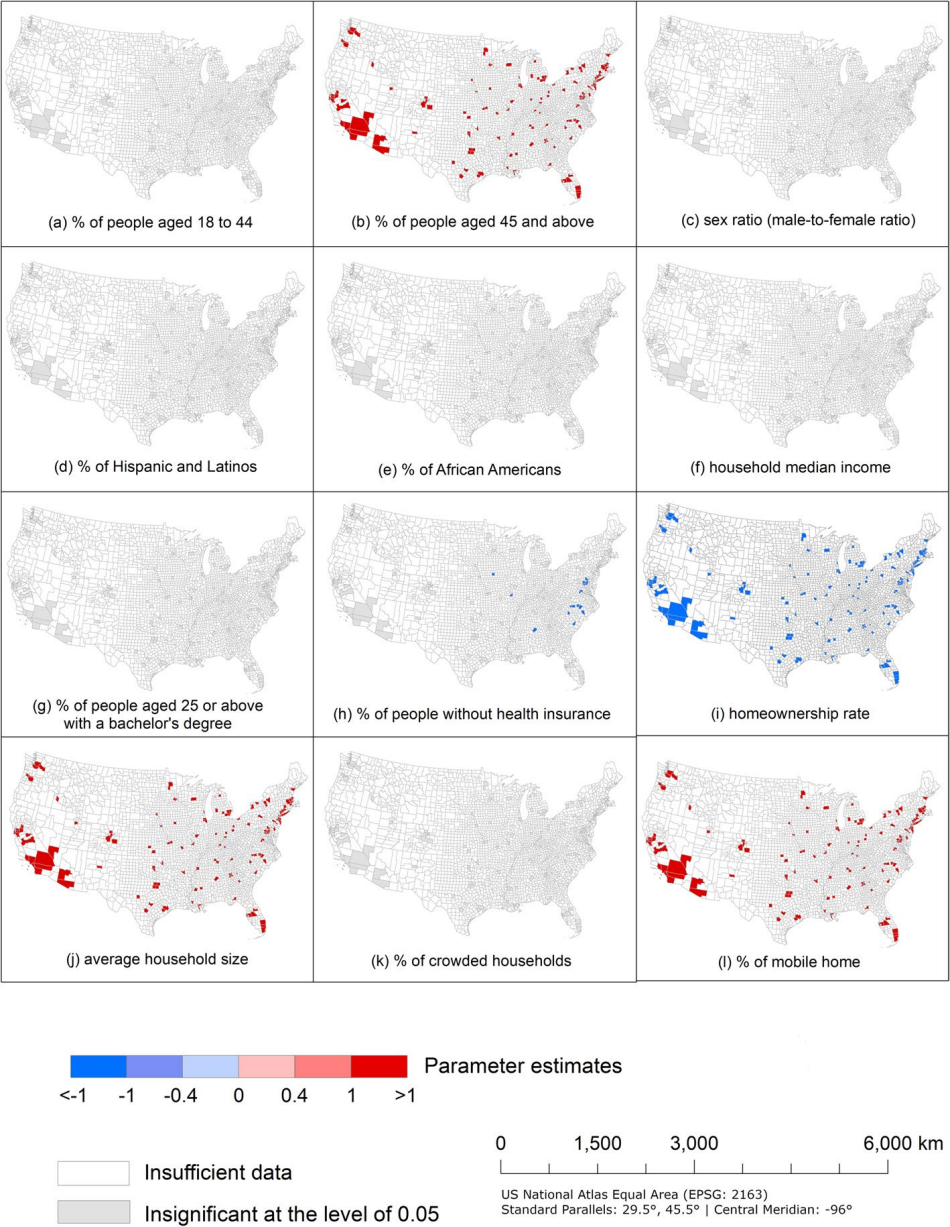
The parameter estimates for associations between percentage of historical-redlining-related geotagged tweets with negative sentiment and shaping factors in U.S. counties based on MGWR model

Figure 3 shows the parameter coefficients and significance of corresponding *t*-values for all 12 explanatory shaping factors in each county based on the results of the MGWR model. Coefficient sign indicates the direction of the relationship, whereas the corresponding *t*-value that is greater than the 95% threshold value indicates statistical significance. Compared with the OLS model, MGWR model shows a better goodness of fit, since the ${R}^{2}$ value increases from 0.114 to 0.285, whereas the corrected AIC value decreases from 452.613 to 446.730.

MGWR results for four potential explanatory shaping factors indicate significant national associations with the percentage of redlining-related geotagged tweets with negative sentiment. Percentage of people aged 45+ (Fig. 3(b)), average household size (Fig. 3(j)), and mobile home percentage (Fig. 3(l)) indicate a statistically significant positive association with public perceptions of redlining practices across all counties included in this study. With the increase of percentage of people aged 45+ , average household size, and mobile home percentage, percentage of redlining-related geotagged tweets with negative sentiment rises. The association between percentage of people aged 45+ and percentage of geotagged tweets with negative sentiment is consistent with literature that redlining practices potentially affect current elder residents’ health behavior as it has a long-term adverse impact on neighborhood environment in former lower-graded HOLC neighborhoods (Besser et al., 2022), possibly impacting their sentiment towards redlining practices. As an example, elder adults living in neighborhoods with HOLC grades C and D reported less walking in their neighborhoods as they had less open park/space than those living in neighborhoods with HOLC grades A and B (Besser et al., 2022).

Similarly, average household size also indicates nationally significant positive associations with percentage of redlining-related geotagged tweets with negative sentiment, which aligns with the result of OLS. One possible reason is that neighborhoods with lower HOLC grades often suffered from systemic disinvestment and concentration of poverty, limiting access to affordable housing (Appel & Nickerson, 2016; Mitchell et al., 2024). Consequently, families or individuals may have to share housing to mitigate costs, leading to larger household sizes. Therefore, household size may potentially fuel public criticism and negative perceptions of redlining practices.

Furthermore, counties with a higher mobile home percentage are also associated with more negative sentiments in redlining-related tweets, and one possible reason is that mobile homes are more common in areas with high poverty rates and limited access to mortgage lending (Kaul & Pang, 2022). This result is consistent with the result based on dataset this study used that there is a higher concentration of mobile homes in lower HOLC-graded neighborhoods. Therefore, people living in mobile homes may view redlining practices as unfair and misaligned with values of equity and opportunity.

However, homeownership rates indicate a significant negative association. Counties with lower homeownership rates are associated with stronger negative sentiment in redlining-related tweets across all counties in this study (Fig. 3(i)). This result is supported by one of the tweets “*This is what current day redlining looks like. Homeownership, known as one of the best ways to build wealth, still preferences whites’ families due to segregation, racism and tax policies that disadvantage Black Americans.*” This idea is further supported by Park and Quercia (2020), who found that redlining practices are associated with lower homeownership rates. This limited access to homeownership has hindered wealth accumulation and contributed to negative sentiments toward redlining practices. Considering the strong positive or negative association of those shaping factors with a coefficient of more than 1 or less than -1, those patterns indicate a spatial stationary across the whole country.

In addition, a significant negative association can be observed between the percentages of populations that are uninsured and the percentage of redlining-related geotagged tweets with negative sentiment in some eastern U.S. counties (Fig. 3(h)). This indicates that areas with higher rates of uninsured individuals are less likely to express negative sentiment in redlining-related tweets. This result contradicts the finding that redlining practices and residential segregation are associated with healthcare and health inequities (Kramer & Hogue, 2009; Williams & Collins, 2001), because residents in neighborhoods of systematically disinvested and lower HOLC grades face many challenges, such as the lack of access to healthcare providers, long travel times for appointments, and the lack of access to private health insurance (Gaskin et al., 2012; Mudd et al., 2019). One possible reason is that these individuals may face barriers like financial constraints or limited access to technology, which can limit their social media engagement and make their views on redlining practices less visible in geotagged tweets (Patagar et al., 2023). Additionally, their focus on immediate concerns, such as healthcare and basic needs, may leave little time to express opinions on redlining practices, contributing to their underrepresentation in online discussions. However, this trend is inconsistent across all U.S. counties in this study, as insignificant associations were detected in other counties.

According to Public Opinion Theory (POT) (Glynn et al., 2015), people form opinions not only from current conditions but also from historical context, lived community experiences, and prevailing social norms. This helps explain why older age, lower homeownership, higher mobile-home rates, and larger household size are associated with more negative sentiment in redlining-related tweets in those counties. Older adults are more likely to recognize that current housing disparities stem from historically entrenched discriminatory systems such as redlining practices rather than merely individual choices, leading counties with a larger older population to exhibit more negative sentiment in redlining-related tweets. Similarly, lower homeownership, more mobile homes, and overcrowding indicate that residents face constrained housing conditions and experience limited opportunities to stable property ownership in their own communities. These lived experiences make historical policies like redlining practices feel more immediate and personally relevant. As a result, people in those counties may be more inclined to express negative sentiment in redlining-related tweets.

As indicted by examination of the maps in Fig. 3, there is no significant association between percentage of redlining-related geotagged tweets with negative sentiment and percentage of people aged 18–44 (Fig. 3(a)), sex ratio (male-to-female ratio) (Fig. 3(c)), percentage of Hispanic and Latinos (Fig. 3(d)), percentage of African Americans (Fig. 3(e)), household median income (Fig. 3(f)), educational attainment (Fig. 3(g)), and percentage of crowded households (Fig. 3(k)). Therefore, those shaping factors were not effective to predict public perceptions of redlining practices.

Bandwidth is the number of closest counties from which the data are used for calculation of each local regression, and this informs us on the role of the spatial scales for the relationship between percentage of redlining-related geotagged tweets with negative sentiment and explanatory shaping factors (Li & Fotheringham, 2020; Oshan et al., 2019). Using the bandwidth, the underlying process of data generation for spatial scales of each shaping factor can be intuitively explained (Li & Fotheringham, 2020; Oshan et al., 2019). Table 4 shows the optimal bandwidths for all potential explanatory shaping factors with the confidence level of 0.05. Considering 157 counties were included in this study, we categorize spatial scales into global scale (> 100 counties borrowed and weighted), regional scale (50–100), and local scale (< 50 counties). As a result, 11 shaping factors indicated national influence on the public perceptions of redlining practices due to bandwidths of 140 to 155. However, no regional influence was observed, as none of the shaping factors exhibited a bandwidth within the range of 50 to 100. Uninsured population percentage has an optimal bandwidth of 44, indicating a local scale of association with public perceptions of redlining practices. In other words, a local spatial stationary influence of this shaping factor was observed in certain counties, but this relationship was not manifested in other counties.

**Table 4:** Optimal bandwidths of all potential explanatory shaping factors based on MGWR model

Explanatory shaping factor	Bandwidth	Confidence interval (95%)
Percentage of people aged 18 to 44	155	(113, 155)
Percentage of people aged 45 and above	155	(113, 155)
Sex ratio (male-to-female ratio)	155	(86, 155)
Percentage of Hispanics and Latinos	155	(113, 155)
Percentage of African Americans	140	(113, 147)
Household median income	140	(113, 147)
Percentage of people aged 25 or above with a bachelor’s degree	155	(113, 155)
Percentage of people without health insurance	44	(44, 69)
Homeownership rate	155	(113, 155)
Average household size	155	(113, 155)
Percentage of crowded households	155	(113, 155)
Percentage of mobile home	155	(86, 155)

Based on the results of OLS and MGWR models, only one explanatory shaping factor’s parameter estimate shows a consistent significant association, which is average household size (Table 3 and Fig. 3). However, three more explanatory shaping factors also demonstrate a significant association nationwide based on MGWR model, which are percentage of people aged 45+, homeownership rates, and mobile home percentage. In addition, uninsured population percentage demonstrates a local statistically significant association in MGWR model with the confidence level of 0.05.

## Limitations and future research

Several limitations of the research warrant some discussion. First, with the limited number of redlining-related geotagged tweets, this study only included 157 counties with 20 or more geotagged tweets related to redlining practices. The selection criteria are a tradeoff between the number of counties and number of tweets in each county, with the purpose of maintaining the robustness of geospatial analyses. We also tested thresholds of 10 and 30 tweets per county. The 10-tweet threshold retains 174 counties and produces comparable model fit (*R*^*2*^ = 30.4%). An excessively limited number of redlining-related geotagged tweets in each county may not adequately represent the broader public perceptions of redlining practices and increase uncertainty and limit reliability for multivariate local modeling. The 30-tweet threshold reduces the samples to only 87 counties, which is insufficient for MGWR due to the small number of spatial units. Although this study excluded some tweet samples, particularly those from counties with fewer than 20 redlining-related geotagged tweets, it does cover more data samples in a larger geographical area over a longer period, in contrast to traditional data collection methods, such as survey and interview.

Second, the study only used X as the data source to explore people’s perceptions of redlining practices. Similar to traditional data collection methods, geotagged tweets may be subject to sampling bias in demographic characteristics since tweets without geotags and non-social media users are excluded from the analysis (Zhang & Zhu, 2018). For instance, it overrepresents younger, urban, and more technologically engaged populations. Because X does not provide individual-level demographic attributes, such as age, gender, and race, it is not feasible to apply weighting procedures or conduct demographic comparisons typically employed in survey research. However, Wang et al. (2015) found that the tweet samples collected through X API preserve sufficient information for research involving sentiment analysis. Therefore, they remain well suited for understanding broader public discourse and collective behavioral responses, but not for demographic profiling or weighted population estimates. A potential future direction involves incorporating data from additional social media platforms, such as Facebook and Instagram. By expanding the scope of data sources, it may be possible to gather a more substantial number of samples, ensuring a more accurate reflection of public perceptions of redlining practices. This approach could also help mitigate potential biases in the current dataset.

Third, the quantity of redlining-related geotagged tweets is both limited and unevenly distributed across years. As a result, this study focused solely on analyzing the overall public perception of redlining practices within the period of 2011 to 2023 from a spatial lens. However, the study did not account for temporal changes in public perspective during this time frame. In the future, researchers could gather more relevant data from various time periods to examine changes in public perceptions of redlining practices. This would be contingent upon the availability of a sufficient number of tweets to ensure meaningful analysis.

Fourth, we deployed the BERTweet-based sentiment analysis model for the sentiment analysis on redlining-related geotagged tweets (Pérez et al., 2021). As helpful as this technique is in exploring sentiment of people sending tweets, it may introduce uncertainty and potential inaccuracies in predictions, because the model's training data includes 850 million general tweets, which are not specifically related to redlining practices. Future research could manually train a sentiment classifier using tweets specifically related to redlining practices, if a sufficient volume of relevant tweets is available. This approach would enhance the accuracy of sentiment analysis and better reflect public perception of redlining practices.

Fifth, ethical considerations remain important, although this study only uses publicly accessible social media data. Tweets are publicly accessible but not created with the expectation of being used for research, raising concerns related to user privacy. To avoid risk, we restricted our analysis to aggregated county-level public sentiment rather than individual accounts, refrained from quoting any identifiable content, and did not collect personal profile information. There is also potential for ecological fallacy resulting from the use of county-level census data to contextualize individual tweets. Since X does not provide detailed demographic information about individual users—such as age, gender, race, or income—this analysis relies on aggregated data from the U.S. Census Bureau at the county level to approximate the social and demographic context in which tweets were posted. However, the characteristics of the population within a county may not accurately represent the individuals who are active on X or who are tweeting about redlining practices. For example, a tweet posted from a county with a high median income does not necessarily reflect the views or circumstances of a high-income individual. As a result, any associations made between tweet content and county-level characteristics must be interpreted with caution. This limitation highlights the broader challenge of using ecological data to infer individual behavior or sentiment. Future studies could address this issue by exploring methods for estimating user-level demographics, while adhering to ethical and privacy standards.

## Conclusion

This study examined public perceptions of historical redlining practices using geotagged tweets from 2011 to 2023 in U.S. counties. The BERTweet-based sentiment analysis model was deployed to detect each tweet’s sentiment. OLS and MGWR models were used to investigate the association between the public perceptions of redlining practices and potential shaping factors. Results of both models show that average household size has a positive association with percentage of redlining-related geotagged tweets with negative sentiment at the national scale. However, MGWR results show that three other potential explanatory shaping factors also suggest a national-scale significant association with public perceptions of redlining practices. In counties with a higher percentage of people aged 45+, a lower homeownership rate, and a higher mobile home percentage, redlining-related tweets with negative sentiment are more prevalent. In addition, some eastern U.S. counties with a higher uninsured population percentage are less likely to express negative sentiment in redlining-related tweets.

The findings of this study can help the public in better understanding and predicting public perceptions of redlining practices through a geographical lens. It can also offer insights into the spatial patterns and influences of various shaping factors on these perceptions. The methodology can also be used to collect, analyze, and predict public perceptions of other controversial social topics, such as siting of landfills or wind turbines, attitudes of the current U.S. housing crisis, and the adoption of electric vehicles.

## Data Availability

Regarding the tweets analyzed, in compliance with Twitter's Developer Agreement and Policy, we are unable to share the full tweet content. However, we are available to provide the corresponding Tweet IDs, which can be used to retrieve the original tweets using authorized tools. Researchers interested in accessing these Tweet IDs can request them from the corresponding author.
